# Identifying heterogeneous health profiles of primary care utilizers and their differential healthcare utilization and mortality – a retrospective cohort study

**DOI:** 10.1186/s12875-019-0939-2

**Published:** 2019-04-23

**Authors:** Shi Yan, Benjamin Jun Jie Seng, Yu Heng Kwan, Chuen Seng Tan, Joanne Hui Min Quah, Julian Thumboo, Lian Leng Low

**Affiliations:** 1 Duke-NUS Medical School, 8 College Road, Singapore, 169857 Singapore; 2 National University of Singapore, 12 Science Drive 2, Singapore, 117549 Singapore; 3 SingHealth Polyclinics, 167 Jalan Bukit Merah, Tower 5, #15-10, Singapore, 150167 Singapore; 4 Department of Family Medicine & Continuing Care, Singapore General Hospital, 20 College Road, Singapore, 169856 Singapore

**Keywords:** Primary care, Latent class analysis, Population segmentation

## Abstract

**Background:**

Heterogeneity of population health needs and the resultant difficulty in health care resources planning are challenges faced by primary care systems globally. To address this challenge in population health management, it is critical to have a better understanding of primary care utilizers’ heterogeneous health profiles. We aimed to segment a population of primary care utilizers into classes with unique disease patterns, and to report the 1 year follow up healthcare utilizations and all-cause mortality across the classes.

**Methods:**

Using de-identified administrative data, we included all adult Singapore citizens or permanent residents who utilized Singapore Health Services (SingHealth) primary care services in 2012. Latent class analysis was used to identify patient subgroups having unique disease patterns in the population. The models were assessed by Bayesian Information Criterion and clinical interpretability. We compared healthcare utilizations in 2013 and one-year all-cause mortality across classes and performed regression analysis to assess predictive ability of class membership on healthcare utilizations and mortality.

**Results:**

We included 100,747 patients in total. The best model (*k* = 6) revealed the following classes of patients: Class 1 “Relatively healthy” (*n* = 58,213), Class 2 “Stable metabolic disease” (*n* = 26,309), Class 3 “Metabolic disease with vascular complications” (*n* = 2964), Class 4 “High respiratory disease burden” (*n* = 1104), Class 5 “High metabolic disease without complication” (*n* = 11,122), and Class 6 “Metabolic disease with multi-organ complication” (*n* = 1035). The six derived classes had different disease patterns in 2012 and 1 year follow up healthcare utilizations and mortality in 2013. “Metabolic disease with multiple organ complications” class had the highest healthcare utilization (e.g. incidence rate ratio = 19.68 for hospital admissions) and highest one-year all-cause mortality (hazard ratio = 27.97).

**Conclusions:**

Primary care utilizers are heterogeneous and can be segmented by latent class analysis into classes with unique disease patterns, healthcare utilizations and all-cause mortality. This information is critical to population level health resource planning and population health policy formulation.

**Electronic supplementary material:**

The online version of this article (10.1186/s12875-019-0939-2) contains supplementary material, which is available to authorized users.

## Background

Primary care provides “an integrated, accessible health care services” for majority of “personal health care needs” [1, 2]. A good primary care system is associated with a more equitable distribution of health in populations [3]. Primary care, as the foundation of the healthcare system, holds great potential to reduce differences in health across population subgroups and improve populations’ overall health [3–5]. In Singapore, the Ministry of Health (MOH) is committed to transforming the healthcare landscape in view of the evolving health care needs of its population in the community setting. This is timely in the background of aging society and increasing healthcare expenditure. MOH Singapore has been working on initiatives to enable the appropriate management of patients in primary care where specialists in hospitals work with primary care physicians to manage patients with stable but complex conditions in a shared care program [6]. As demand for primary care and complexity in population health needs are growing, primary care systems globally face tremendous challenges. One of the notable challenges is the heterogeneity in population health needs and the resultant difficulty in health care resources planning [7]. To address this challenge in population health management in primary care setting, it is critical to have a better understanding of primary care utilizers’ heterogeneous health state profiles.

Population segmentation is an emerging approach that aims to address this issue. It aims to divide a patient population with heterogeneous heath profiles into distinct and relatively homogenous subgroups (classes) that share similar healthcare needs [8–10]. It enables development of targeted healthcare interventions for each population segment and facilitates healthcare resource planning [11, 12]. Population segmentation frameworks have been widely applied to provide quantitative overviews of population health characteristics and guide population health policy and resource management. For example, Ministry of Health British Columbia, Canada adopted a population segmentation framework dividing the entire British Columbia provincial population into 13 classes that represented different health status and healthcare needs [13].

Recently, data-driven population segmentation that utilizes post-hoc statistical analysis on empirical data is gaining wide interest worldwide. It utilizes large volumes of patients’ data to support population health policy decisions by generating real-life, evidence-based, and quantitative insights of a population’s health status [14]. The rich healthcare data made accessible by adoption of electronic health records globally provide opportunities for population segmentation analysis using empirical data [15]. Additionally, the recent advancement in big data analytics in population health management allows for more computational tools for accurate population segmentation. As an example, latent class analysis by Van der Laan et al. on self-reported data successfully segmented an elderly population into classes with different healthcare needs and demonstrated differential healthcare service utilization patterns in different classes [16]. To date, data-driven population segmentation has been used on wide range of populations, including geriatric [17], pediatric [18, 19] population, and gynecological [20], respiratory [21], and oncological patients [22]. However, to the best of authors’ knowledge, data-driven population segmentation has not been applied to primary care utilizers.

The primary aim of this study is to segment a population of primary care utilizers into classes of unique disease patterns, and to report the disease patterns, one-year follow up healthcare utilizations and all-cause mortality across the classes. The secondary aim is to assess the predictive ability of class membership on one-year follow up healthcare utilizations and mortality.

## Methods

### Study design

In this retrospective cohort study, we retrieved de-identified administrative health data from the population health database at Singapore Health Services Regional Health System (SingHealth RHS), the Singapore’s largest RHS that provides comprehensive care in its primary care clinics, community hospitals, national specialty centers and tertiary hospitals for a specific geographic region. The data included in this study are patients’ baseline demographics, disease diagnosis according to International Classification 9 and 10 codes, and longitudinal data on healthcare utilizations (number of inpatient admissions to hospitals and visits to emergency departments, specialist outpatient clinic, and primary care clinics) in 2013, and one-year all-cause mortality. Inpatient admissions refer to patient visits to the SingHealth hospitals that culminated in patients being hospitalized and day surgeries were not included. Primary care visit was defined as a visit to a SingHealth primary care facility (polyclinics) and specialist outpatient clinic as a visit to a hospital specialist clinic respectively. Telephone visits were not included.

The inclusion criteria are 1) adult patients above 21 years old (age of majority in Singapore), and 2) Singapore citizens or permanent residents, and 3) utilized services in SingHealth RHS primary care clinics in Year 2012. Charlson Comorbidity Index [23], Elixhauser Index [24] and Singapore Chronic Disease Management Program [25] was used to select the chronic diseases included in this study. For diseases that had overlap between Charlson Comorbidity Index and Elixhauser Index, diseases coded in the latter index was utilized as they have been shown to provide better prediction of healthcare utilization and mortality [26, 27]. We excluded patients whose residential postal codes fall outside SingHealth RHS catchment region so as to reflect health utilization patterns accurately because these patients may tend to have care utilizations outside SingHealth RHS. The SingHealth Centralized Institutional Review Board (reference number: CIRB 2016/2294) issued the ethical approval for this study.

### Latent class analysis (LCA)

LCA is a model-based tool which is widely used to identify unobserved (latent) subgroups amongst heterogeneous population [28]. LCA as a person-centered approach aims to divide individuals into categories, with individuals in the same category being relatively homogeneous, and at the same time distinct from those in other categories [16, 29]. LCA estimates two parameters based on maximum-likelihood: 1) class membership probabilities, which represent individuals’ probability of belonging to each class, and 2) item-response probabilities conditional on class membership (conditional response probabilities), which refer to the conditional probability a particular response given the individual is in a certain class [30–32]. Based on their highest latent class probability, individuals are assigned to one class exclusively. Within each class, individuals have similar conditional item response probability patterns [31, 33].

The latent classes derived from LCA can reflect many aspects of health, depending on the class indicators used. Here we focus on population health state profiles in primary care setting and thus choose to use chronic disease status as class indicators.

Mplus version 8 statistical modeling software was used for conducting LCA [28].

### Model selection

We fit LCA successively from k = 2 onwards (k is the desired number of classes) and stopped the succession when a class size of a particular model is less than 1 % of the population. Each class should have a substantial size (≥1 % of the population) so that it can be targeted with distinctive heath intervention strategies at policy level. We assessed model fit using multiple criteria. Firstly, established statistical indexes have been widely used such as Akaike Information Criteria (AIC) and Bayesian Information Criterion (BIC) where a smaller AIC and BIC indicates a better fit [34–36]. Secondly, in order to have clinical relevance, the model has to have clinical interpretability. Clinical interpretability of classes was evaluated through the integration of clinical expert knowledge and existing clinical guidelines, which are likely to predict differences in healthcare utilization and outcomes [37, 38].

### Statistical analysis

Firstly, to examine whether significant cross-class differences in disease diagnosis patterns, demographics and healthcare utilization in baseline Year 2012 exist, we used one-way ANOVA test (or Kruskal-Wallis H test with Bonferroni correction) for continuous variables and Chi-square test (or Fisher exact test) for categorical variables as appropriate.

Then, we assessed the discriminative properties of class membership on healthcare utilizations and mortality in 2013. We began by excluding patients who deceased within 2012 because in 2013 they would have no healthcare utilization (*n* = 761). We then ran Kruskal-Wallis H test and Chi-square test between the the healthcare utilization (nonparametric) and mortality in 2013 and population classes respectively. When it came to count variable outcomes (e.g., one-year follow up healthcare utilization), to examine the relationship between healthcare utilization and class membership in 2013, we conducted a multivariable analysis via Poisson or negative binomial regression (with the use of the offset/exposure option) where appropriate. The class membership is the exposure of interest adjusting for ethnicity, age, and gender [9]. In anticipation of people who would die, offset term was used, which is the log of the follow-up time starting from 01 Jan 2013 ending on 1) 31 Dec 2013 for participants who lived beyond 1 Jan 2014 or 2) the death date for those who died before 31 Dec 2013. We performed multivariable Cox proportional hazard regression analysis to examine the relationship of class membership and mortality rate. We also presented Hazard Ratio (HR) and its 95% confidence interval. The models were adjusted for age, gender, and ethnicity. Lastly, we used Kaplan Meier estimator for the survival function from lifetime data. Log-rank test was used to compare the differences of survival distributions between the classes. Kaplan-Meier survival curves for one-year mortality (Year 2013) were plotted with 01 January 2013 as time of entry into the follow up period. The time to survival was defined as the number of days from 01 January 2013 to death or 365 days for patients who are deceased on/before 31 December 2013 and censored patients who lived beyond 2013, respectively. STATA SE 14.0 (Stata Corporation, College Station, Texas, 2016) was used for all the analysis.

## Results

Patient demographics in baseline Year 2012.

We included 100,747 patients in this study. Table 1 shows the disease prevalence and healthcare utilization of patients in baseline Year 2012. Patients’ mean age is 51.7 ± 17.4 years old. 45.2% (*n* = 45,515) patients were male. Majority of patients were of Chinese ethnicity (*n* = 78,414, 77.8%).

**Table 1:** Baseline demographics, clinical characteristics and healthcare utilization of patients in Year 2012

Characteristics ^a^	Class 1 Relatively healthy (*n*=58,213, 57.8%)	Class 2 Stable metabolic disease (*n*=26,309, 26.1%)	Class 3 Metabolic disease with vascular complications (*n*=2,964, 3.0%)	Class 4 High respiratory disease burden (*n*=1,104, 1.1%)	Class 5 High metabolic disease without complication (*n*=11,122, 11.0%)	Class 6 Metabolic disease with multi-organ complication (*n*=1,035, 1.0%)	Overall (*n*=100,747)	*p*-value
Age, (SD)	43.1 (15.0)	62.1 (12.2)	72.9 (11.1)	54.2 (19.4)	64.8 (11.5)	70.6 (13.3)	51.7 (17.4)	<0.001
Gender								
Male, (%)	25,979 (44.6)	11,399 (43.3)	1,620 (54.7)	538 (48.7)	5,392 (48.5)	587 (56.7)	45,515 (45.2)	<0.001
Race								
Chinese, (%)	42,890 (73.7)	22,596 (85.9)	2,468 (83.3)	687 (62.2)	8,971 (80.7)	802 (77.5)	78,414 (77.8)	<0.001
Malay, (%)	8.258 (14.2)	1,635 (6.2)	174 (5.9)	208 (18.8)	991 (8.9)	116 (11.2)	11,382 (11.3)	
Indian, (%)	4,511 (7.8)	1,520 (5.8)	248 (8.4)	171 (15.5)	889 (8.0)	91 (8.8)	7,430 (7.4)	
Others, (%)	2,554 (4.4)	558 (2.1)	74 (2.5)	38 (3.4)	271 (2.4)	26 (2.5)	3,521 (3.5)	
Social determinants of health								
Public rental housing, (%)	4,406 (7.6)	1,929 (7.3)	401 (13.5)	275 (24.9)	949 (8.5)	162 (15.7)	8,122 (8.1)	
Comorbidities								
Type 2 Diabetes mellitus (%)	451 (0.8)	4,981 (18.9)	1,592 (53.7)	175 (15.9)	11,122 (100)	654 (63.2)	18,975 (18.8)	<0.001
Hypertension, (%)	3,883 (6.7)	19,099 (72.6)	2,881 (97.2)	432 (39.1)	11,040 (99.3)	970 (93.7)	38,305 (38.0)	<0.001
Hyperlipidemia, (%)	2 (0.01)	23,427 (89.1)	2,809 (94.8)	334 (30.3)	11,060 (99.4)	865 (83.6)	38,497 (38.2)	<0.001
Type 2 diabetes mellitus with complication, (%)	23 (0.04)	0 (0)	103 (3.5)	5 (0.5)	1,586 (14.3)	205 (19.8)	1,922 (1.9)	<0.001
Chronic kidney disease stage 3 and 4, (%)	50 (0.1)	945 (3.6)	239 (8.1)	12 (1.1)	1,408 (12.7)	1,035 (100)	3,689 (3.7)	<0.001
Chronic kidney disease stage 5, end stage renal disease, (%)	0 (0)	0 (0)	0 (0)	1 (0.1)	0 (0)	1,003 (96.9)	1,004 (1.0)	<0.001
Coronary artery disease, (%)	198 (0.3)	2,433 (9.3)	2,485 (83.8)	53 (4.8)	1,954 (17.6)	523 (50.5)	7,646 (7.6)	<0.001
Atrial fibrillation, (%)	45 (0.1)	99 (0.4)	514 (17.3)	20 (1.8)	1 (0.01)	139 (13.4)	818 (0.8)	<0.001
Heart failure, (%)	88 (0.2)	114 (0.4)	863 (29.1)	32 (2.9)	15 (0.1)	322 (31.1)	1,434 (1.4)	<0.001
Peripheral vascular disease, (%)	24 (0.04)	95 (0.4)	372 (12.6)	9 (0.8)	132 (1.2)	117 (11.3)	749 (0.7)	<0.001
Stroke, (%)	145 (0.3)	1,565 (6.0)	1,232 (41.6)	32 (2.9)	819 (7.4)	282 (27.3)	4,075 (4.0)	<0.001
Asthma, (%)	1,774 (3.1)	777 (3.0)	423 (14.3)	901 (81.6)	314 (2.8)	95 (9.2)	4,284 (4.3)	<0.001
Chronic obstructive pulmonary disease, (%)	210 (0.4)	99 (0.4)	564 (19.0)	1,096 (99.3)	74 (0.7)	144 (13.9)	2,187 (2.2)	<0.001
Depression, (%)	1,112 (1.9)	713 (2.7)	195 (6.6)	79 (7.2)	64 (0.6)	79 (7.6)	2,242 (2.2)	<0.001
Dementia, (%)	17 (0.03)	91 (0.4)	135 (4.6)	5 (0.5)	56 (0.5)	49 (4.7)	353 (0.4)	<0.001
Anxiety, (%)	545 (0.9)	361 (1.4)	66 (2.2)	31 (2.8)	18 (0.2)	21 (2.0)	1,042 (1.0)	<0.001
Osteoarthritis, (%)	4,870 (8.4)	7,716 (29.3)	737 (24.9)	220 (19.9)	689 (6.2)	288 (27.8)	14,520 (14.4)	<0.001
Benign prostate hyperplasia, (%)	189 (0.3)	454 (1.7)	208 (7.0)	18 (1.6)	25 (0.2)	27 (2.6)	921 (0.9)	<0.001
Hyperthyroidism, (%)	526 (0.9)	442 (1.7)	33 (1.1)	18 (1.6)	19 (0.2)	6 (0.6)	1,044 (1.0)	<0.001
Hypothyroidism, (%)	453 (0.8)	1,127 (4.3)	98 (3.3)	29 (2.6)	87 (0.8)	27 (2.6)	1,821 (1.8)	<0.001
Malignancy, (%)	627 (1.1)	1,137 (4.3)	298 (10.1)	79 (7.2)	387 (3.5)	91 (8.8)	2,619 (2.6)	<0.001
Metastatic disease, (%)	90 (0.2)	168 (0.6)	56 (1.9)	11 (1.0)	12 (0.1)	11 (1.1)	348 (0.4)	<0.001
Healthcare utilization in Year 2012								
Number of primary care outpatient clinic visits, (SD)	2.8 (4.0)	5.7 (3.8)	6.7 (5.7)	5.6 (10.3)	6.6 (4.5)	6.5 (8.7)	4.1 (4.6)	<0.001
Number of outpatient specialist clinic visit, (SD)	1.7 (4.3)	2.8 (6.0)	5.0 (7.8)	4.2 (8.6)	2.5 (5.4)	10.0 (12.5)	2.7 (5.4)	<0.001
Number of hospital admission, (SD)	0.1 (0.4)	0.1 (0.5)	0.5 (1.2)	0.4 (0.9)	0.1 (0.5)	1.2 (1.8)	0.1 (0.5)	<0.001
Number of emergency department visits, (SD)	0.1 (0.5)	0.1 (0.5)	0.2 (0.6)	0.6 (1.5)	0.2 (0.6)	1.2 (1.9)	0.2 (0.7)	<0.001

Abbreviations: *SD* standard deviation

^a^Kruskal Wallis test or ANOVA test was used to compare healthcare utilization between the 6 classes while Chi-Square test was used to compare the mortality data among the classes

### Latent class model selection

For the latent class selection, the LCA analyses was run from k = 2 to k = 8. However, for k = 7 and k = 8, some of the class sizes fell below 1% of the population. Hence, further statistical analyses were only performed for k = 2 to k = 6.

**Fig. 1:**
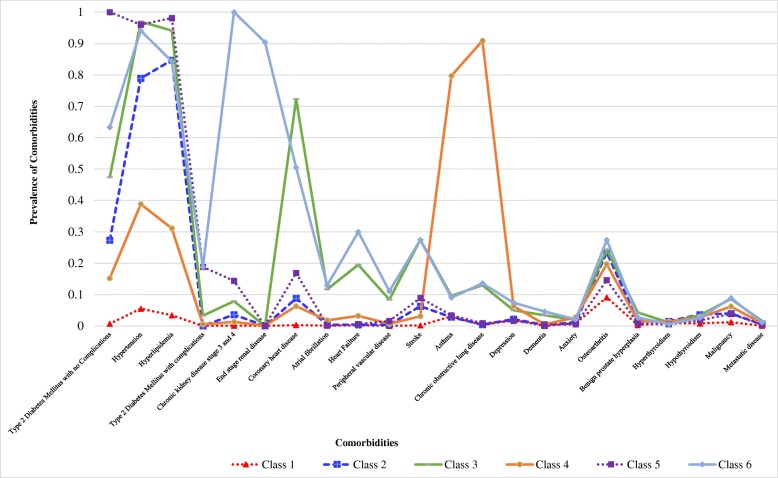
Graphical display of comorbidities of patients by latent classes (k = 6)

A six-class model was selected for interpretation based on its better statistical fit as suggested by lowest AIC and BIC (Table 2). Figure 1 depicts the graphical representation of disease patterns across the six classes. The prevalence of diseases was generally low in patients in Class 1. Patients in Class 2 and 3 had higher prevalence of hypertension and hyperlipidemia. The prevalence of peripheral vascular disease, stroke, and coronary artery disease, were the highest in Class 3 patients. The prevalence of asthma and chronic obstructive pulmonary disease were the highest among Class 4 patients. Prevalence of metabolic diseases among Class 5 and 6 patients such as diabetes mellitus, hypertension and hyperlipidemia were high. Class 6 patients had higher prevalence of diabetes mellitus with complications, chronic kidney disease, heart failure and vascular complications such as peripheral vascular disease, stroke, and coronary artery disease. Hence, the six classes were named: Class 1 “Relatively healthy”, Class 2 “Stable metabolic disease”, Class 3 “Metabolic disease with vascular complication”, Class 4 “High respiratory disease burden”, Class 5 “High metabolic disease without complication” and Class 6 “Metabolic disease with multi-organ complications”.

**Table 2:** Criteria to assess model fit for latent class analysis models

Number of Classes (k)	Class sizes	Akaike (AIC)	Bayesian (BIC)	Sample-Size Adjusted BIC
2	Class 1 = 65,012 (64.5%) Class 2 = 35,735 (35.5%)	672,123	672,551	672,409
3	Class 1 = 64,813 (64.3%) Class 2 = 32,519 (32.3%) Class 3 = 3415 (3.4%)	662,674	663,321	663,105
4	Class 1 = 64,091 (63.6%) Class 2 = 32,389 (32.2%) Class 3 = 3013 (3.0%) Class 4 = 1254 (1.2%)	658,999	659,866	659,576
5	Class 1 = 61,792 (61.3%) Class 2 = 24,225 (24.1%) Class 3 = 2510 (2.5%) Class 4 = 1209 (1.2%) Class 5 = 11,011 (10.9%)	656,030	657,115	656,753
6	Class 1 = 58,213 (57.8%) Class 2 = 26,309 (26.1%) Class 3 = 2964 (2.9%) Class 4 = 1104 (1.1%) Class 5 = 11,122 (11.0%) Class 6 = 1035 (1.0%)	653,089	654,394	653,958

### Healthcare utilization and all-cause mortality in follow-up year 2013

Table 3 shows the healthcare utilization and all-cause mortality among patients in the six classes in Year 2013. Class 6 “Metabolic disease with multi-organ complications” patients had the highest number of outpatient specialist clinic and emergency department visits and hospital admissions (*p* < 0.001). Additionally, they had the highest all-cause mortality. (p < 0.001).

**Table 3:** Healthcare utilization patients in 2013 and one-year all-cause mortality (k = 6)

Healthcare utilization / mortality ^a^	Class 1 Relatively healthy	Class 2 Stable metabolic disease	Class 3 Metabolic disease with vascular complications	Class 4 High respiratory disease burden	Class 5 High metabolic disease without complication	Class 6 Metabolic disease with multi-organ complication	Overall	*p*-value
Number of primary care outpatient clinic visits, (SD)	2.0 (3.9)	5.3 (3.9)	6.0 (5.5)	4.7 (6.0)	6.2 (4.2)	4.9 (7.6)	3.5 (4.4)	< 0.001
Number of outpatient specialist clinic visit, (SD)	1.6 (4.6)	2.7 (5.9)	4.7 (7.8)	3.8 (7.5)	2.6 (5.7)	8.6 (11.9)	2.2 (5.5)	< 0.001
Number of hospital admission, (SD)	0.1 (0.4)	0.1 (0.5)	0.5 (1.1)	0.3 (0.9)	0.1 (0.5)	1.0 (1.7)	0.1 (0.5)	< 0.001
Number of emergency department visits, (SD)	0.1 (0.5)	0.1 (0.6)	0.5 (1.8)	0.6 (2.0)	0.2 (0.6)	1.0 (1.8)	0.1 (0.7)	< 0.001
One-year all-cause mortality	179 (0.3)	207 (0.8)	139 (4.7)	19 (1.7)	125 (1.1)	92 (8.9)	761 (0.8)	< 0.001

*Abbreviations*: *SD* standard deviation

^a^Kruskal Wallis test or ANOVA test was used to compare healthcare utilization between the 6 classes while Chi-Square test was used to compare the mortality data among the classes

### Multivariable analyses of classes and healthcare utilization and mortality in follow-up year 2013

As shown in Table 4, Class 1 “Relatively healthy” was used as the reference group in the multivariable analyses. After adjusting for age, gender, ethnicity, Class 6 “Metabolic disease with multi-organ complications” patients had significantly higher utilization of outpatient specialist clinic (Incidence rate ratio (IRR): 6.60, 95% Confidence Interval (CI): 5.75–7.56), hospital admissions (IRR: 19.68, 95% CI: 16.41–23.61), emergency department visits (IRR: 13.86, 95% CI: 11.74–16.37). Patients in Class 3 “Metabolic disease with vascular complication” and Class 5 “High metabolic disease without complication” had the highest utilization of primary care outpatient clinics (*p* < 0.001). Class 6 “Metabolic disease with multi-organ complications” patients had the highest risk of all-cause mortality (Hazard ratio (HR): 27.97, 95% CI: 25.01–31.29), followed by patients in Class 3 “Metabolic disease with vascular complication” (HR: 14.57, 95% CI: 13.25–16.01) (Table 4).

**Table 4:** Multivariable negative binomial regression on healthcare utilization and cox proportional hazards regression on mortality in Year 2013 (k = 6)

Healthcare utilization or mortality ^a^	IRR, unless otherwise specified	95% Confidence interval	p-value
Number of primary care outpatient clinic visits			
Class 1	1.00	*Reference*	
Class 2	2.69	2.64–2.73	< 0.001
Class 3	3.20	3.08–3.32	< 0.001
Class 4	2.42	2.28–2.57	< 0.001
Class 5	3.16	3.09–3.22	< 0.001
Class 6	2.83	2.66–3.02	< 0.001
Number of outpatient specialist clinic visit			
Class 1	1.00	*Reference*	
Class 2	1.67	1.62–1.74	< 0.001
Class 3	3.33	3.07–3.62	< 0.001
Class 4	2.60	2.28–2.98	< 0.001
Class 5	1.68	1.60–1.76	< 0.001
Class 6	6.60	5.75–7.56	< 0.001
Number of hospital admission			
Class 1	1.00	*Reference*	
Class 2	1.75	1.64–1.86	< 0.001
Class 3	8.05	7.14–9.07	< 0.001
Class 4	4.79	3.93–5.83	< 0.001
Class 5	2.20	2.03–2.38	< 0.001
Class 6	19.68	16.41–23.61	< 0.001
Number of emergency department visits			
Class 1	1.00	*Reference*	
Class 2	1.66	1.57–1.75	< 0.001
Class 3	6.89	6.19–7.67	< 0.001
Class 4	6.68	5.65–7.89	< 0.001
Class 5	1.91	1.78–2.06	< 0.001
Class 6	13.86	11.74–16.37	< 0.001
One-year all-cause mortality ^b^			
Class 1	1.00	*Reference*	
Class 2	2.81	2.59–3.04	< 0.001
Class 3	14.57	13.25–16.01	< 0.001
Class 4	7.03	5.86–8.42	< 0.001
Class 5	4.43	4.05–4.83	< 0.001
Class 6	27.97	25.01–31.29	< 0.001

*Abbreviations: IRR* Incidence rate ratio (number of events divided by the person-time at risk)

^a^– Class 1: Relatively Healthy; Class 2: Stable metabolic disease, Class 3: Metabolic disease with vascular complications, Class 4: High respiratory burden, Class 5: High Metabolic disease without complication, Class 6: Metabolic disease with end-organ failure

^b^- Hazard ratio was reported

Models are adjusted for age, gender, and ethnicity. Survival time was used as exposure variable for negative binomial regression

### Analysis of one-year survival time

The Kaplan Meier curve was constructed for all-cause mortality stratified by latent classes (Additional file 1). The one-year mortality of patients in Class 2 to Class 6 were significantly higher than Class 1 patients (p < 0.001), with Class 6 “Metabolic disease with multi-organ complications” patients having the highest one-year mortality rate.

Results for k = 2 to 5 were shown in Additional file 2.

## Discussion

Using latent class analysis, we successfully segmented the heterogeneous population of primary care utilizers into six patient classes with distinct disease patterns. We also demonstrated the derived classes have predicative ability on mortality amd long term healthcare utilization. This supports the feasibility of applying a data-driven population segmentation technique in primary care setting.

Our study provides a detailed and quantitative overview of health status of a large population of primary care users. It can enable health policy makers to make informed decisions on the development of targeted health interventions for each unique. For example, a large proportion of primary care users (57.8%) in our study belong to “Relatively healthy” class and have limited healthcare utilizations (Class 1). For this large segment, health strategies should focus on disease prevention and health promotion. This informs allocation of appropriate health resources to the development of health promotion and education programs as well as preventive services such as screening tests by community-based service providers [14, 39, 40]. For the “Stable metabolic disease” group (Class 2) and “High metabolic disease without complication” (Class 5), health service planning should focus on patients’ disease management education, self-motivation and appropriate clinical monitoring to maintain adequate control of chronic diseases and delay (or prevent) subsequent complications. For the higher utilizing, complex segment of metabolic disease with vascular or multiple organ complications (Class 3 and 6), shared care with appropriate specialists and/or team-based care with community case coordinators are probably required to address the multiple determinants of health and optimize quality of life. One of the useful approaches is a six-step process involving needs assessment, definition of proximal program objective matrices, selection of theory based methods and practical strategy, production of program components and design, program adoption and implementation plan, and finally evaluation plan [41].

Data-driven population segmentation approach is gaining momentum as it leverages on large volumes of empirical healthcare data to generates quantitative and real life insights of population health that supports evidence-based population health policy [14]. With the rapid adoption and expansion of electronic health records globally, data-driven population segmentation has been applied in wide range of populations. For example, Vuik et al. recently demonstrated that data-driven segmentation could be used on a general patient population’s data from healthcare administrative databases [14]. However, despite its wide application in health science and policy literature, no previous study examined primary care users by data-driven population segmentation. To the best of our knowledge, our study is the first to address this critical gap in primary care literature using large scale disease, long term healthcare utilization and mortality data.

Compared to prior studies on segmentation of general population, our segmentation solution generated different population segments. For example, Lafortune et al. used LCA to segment a general elderly population and identified four health state profiles: “Relatively healthy”, “Cognitively Impaired”, “Physically impaired” and “Cognitively and physically impaired” [42]. The differences between the present segmentation solution and the prior studies might be explained by different segmenting variables used for LCA in the present study. In our study, we segmented by disease status to derive different multi-morbidity patterns that are validated by healthcare utilization whereas Lafortune et al. [42] and Liu et al. [29] defined the segments by additional sensory, cognitive and functional data. The different choice of segmenting variables will inevitably result in different definitions and naming of derived segments. Selecting segmenting variables requires careful considerations of clinical significance, policy relevance, and data availability. Our study adds to previous work by discovering the patterns of multi-morbidity that contribute to differential healthcare utilization and mortality. We also observed that mental health diseases such as dementia, depression, and anxiety have low prevalence amongst primary care utilizers in Singapore compared to other disease. This may be multifactorial due to lower prevalence of mental health disease in Asia compared to Western countries [43], biased diagnosis and reporting of mental health disease as a result of cross-cultural application of criteria such as the American Psychiatric Association’s Diagnostic and Statistical Manual [44], and/or mental illness patients’ preference to utilize psychiatrists’ specialist services as opposed to primary care providers’. This deserves future research efforts in understanding their health behavioral preferences and patterns.

Selection of the most appropriate segmentation solution is a complex process and requires interplay of subject matter expertise and data analytics. In the present study, we assessed each segmentation model for scientific robustness and practical utility and implications at population health policy level [45]. First of all, data-driven segmentation solution must be assessed by its statistical fit. In LCA, established diagnostic indexes include Akaike Information Criterion (AIC) and Bayesian Information Criterion (BIC) [36, 46–48]. On top of the basic statistical fit, additional criteria are required to assess its relevance in a particular healthcare system. Currently, the criteria for optimal segmentation framework in population health have not been established [49, 50]. In consumer market segmentation, the segmentation effectiveness is assessed by the following proposed criteria, which could be adopted in healthcare settings: validity, interpretability, substantiality, stability, and actionability/accessibility [51, 52]. Other additional criteria, such as parsimony of number of classes may be important to ensure easy use and widespread adoption of a segmentation framework. Additionally, the naming of each segment is a subjective process in a way which best represented the features of a segment. This may depend on clinical expertise of researchers as well as policy context [9].

One of the limitations of this study is that data were collected from a single cluster of health service institutions (SingHealth RHS). Health services utilizations from non SingHealth RHS were currently not captured in the current database. By excluding resident population whose postal codes fall outside SingHealth RHS catchment region because they are more likely to utilize services outside SingHealth RHS, we attempted to minimize this limitation. Future research can expand to national level data or linking databases from other health services institutions to assess the external validity of our segmentation framework. Some large segments may still have certain degree of heterogeneity which can be further segmented. The current study provides an initial broad segment archetype that can be further refined by additional indicators such as behavioral risk factors, mental health, frailty and social functioning. Another limitation is the relatively short follow-up period. Long-term healthcare utilization and mortality patters of the derived patient segments have important implications in health policy making. Further research efforts may focus on evaluating the long-term stability of the derived patient segments.

## Conclusions

In conclusions, primary care users have heterogeneous health state profiles. They can be segmented into classes with unique, relatively homogeneous health characteristics using latent class analysis. Different classes have different health services utilization patterns and mortality risks. This information is critical to population level health resource planning and population health policy formulation.

## Additional files

Additional file 1: Kaplan Meier survival estimate by patient latent class (k = 6). This file includes the Kaplan Meier survival estimate by patient latent class for all six patient classes. (DOCX 19 kb)

Additional file 2: This file includes results for different models k = 2, 3, 4, and 5. (DOCX 84 kb)

## Abbreviations

AIC: Akaike Information Criterion
BIC: Bayesian Information Criterion
LCA: Latent Class Analysis
SingHealth RHS: Singapore Health Services Regional Health System
