# Supplementary material for: Identifying heterogeneous health profiles of primary care utilizers and their differential healthcare utilization and mortality – a retrospective cohort study
Source: BMC Fam Pract. 2019 Apr 23;20:54. doi: 10.1186/s12875-019-0939-2 (PMC6477732; doi:10.1186/s12875-019-0939-2)
Supplement: Supplementary file 1 — Kaplan Meier survival estimate by patient latent class (k = 6). This file includes the Kaplan Meier survival estimate by patient latent class for all six patient classes. (DOCX 19 kb) [file 12875_2019_939_MOESM1_ESM.docx]

**Kaplan Meier survival estimate by patient latent class (k=6)**

| **Days** | **0** | **100** | **200** | **300** | **365** |
| --- | --- | --- | --- | --- | --- |
| Number at Risk |  |  |  |  |  |
| Class 1: Relatively healthy | 58,213 | 58,009 | 57,963 | 57,920 | 57,892 |
| Class 2: Stable  metabolic disease | 26,309 | 26,121 | 26,051 | 25,994 | 25,971 |
| Class 3: Metabolic disease with vascular complications | 2,964 | 2,828 | 2,791 | 2,762 | 2,736 |
| Class 4: High respiratory disease burden | 1,104 | 1,064 | 1,061 | 1,056 | 1,055 |
| Class 5: High metabolic disease without complication | 11,122 | 10,993 | 10,959 | 10,929 | 10,908 |
| Class 6: Metabolic disease with multi-organ complication | 1,035 | 927 | 902 | 879 | 864 |

Log-Rank Test: p<0.001
