# Supplementary material for: Identifying heterogeneous health profiles of primary care utilizers and their differential healthcare utilization and mortality – a retrospective cohort study
Source: BMC Fam Pract. 2019 Apr 23;20:54. doi: 10.1186/s12875-019-0939-2 (PMC6477732; doi:10.1186/s12875-019-0939-2)
Supplement: Supplementary file 2 — This file includes results for different models k = 2, 3, 4, and 5. (DOCX 84 kb) [file 12875_2019_939_MOESM2_ESM.docx]

**Figure 1a**. Graphical display of comorbidities of patients by Class (k=2)

**Figure 1b**. Graphical display of comorbidities of patients by Class (k=3)

**Figure 1c**. Graphical display of comorbidities of patients by Class (k=4)

**Figure 1d**. Graphical display of comorbidities of patients by Class (k=5)

**Table 2a. Healthcare Utilization of patients in 2013 and 3-year mortality data (k=2)**

| **Healthcare utilization / mortality by year^#^** | **Class 1**  Low comorbidity  **(n=65012)** | **Class 2**  High comorbidity  **(n=35735)** | **Overall**  **(n=100747)** | **p-value** |
| --- | --- | --- | --- | --- |
| Number of primary care outpatient clinic visits, (SD) | 2.2 (3.9) | 5.8 (4.3) | 3.5 (4.4) | <0.001 |
| Number of outpatient specialist clinic visit, (SD) | 1.7 (4.8) | 3.1 (6.5) | 2.2 (5.5) | <0.001 |
| Number of hospital admission, (SD) | 0.1 (0.4) | 0.2 (0.7) | 0.1 (0.5) | <0.001 |
| Number of emergency department visits, (SD) | 0.1 (0.5) | 0.2 (0.8) | 0.1 (0.7) | <0.001 |
| 3 year all-cause mortality, (%) | 841 (1.3) | 2283 (6.4) | 3124 (3.1) | <0.001 |

**Abbreviations**: SB – standard deviation

^#^ Continuous variables were analyzed using Student’s 𝑡-test and categorical variables were analysed using chi-square test or Fisher’s exact test when appropriate

* p-value <0.05

**Table 2b. Healthcare Utilization of patients in 2013 and 3-year mortality data (k=3)**

| **Healthcare utilization / mortality by year^#^** | **Class 1**  Low comorbidity  **(n=64,813)** | **Class 2**  Moderate metabolic disease  **(n=32,519)** | **Class 3**  High metabolic and vascular disease  **(n=3,415)** | **Overall**  **(n=100,747)** | **p-value** |
| --- | --- | --- | --- | --- | --- |
| Number of primary care outpatient clinic visits, (SD) | 2.2 (3.9) | 5.8 (4.0) | 5.7 (6.4) | 3.5 (4.4) | <0.001 |
| Number of outpatient specialist clinic visit, (SD) | 1.7 (4.8) | 2.7 (5.9) | 6.4 (9.8) | 2.2 (5.5) | <0.001 |
| Number of hospital admission, (SD) | 0.1 (0.4) | 0.1 (0.5) | 0.6 (1.4) | 0.1 (0.5) | <0.001 |
| Number of emergency department visits, (SD) | 0.1 (0.5) | 0.2 (0.6) | 0.7 (1.9) | 0.1 (0.7) | <0.001 |
| 3 year all-cause mortality, (%) | 852 (1.3) | 1484 (4.6) | 788 (23.1) | 3124 (3.1) | <0.001 |

**Abbreviations**: SB – standard deviation

^#^ Continuous variables were analyzed using Anova test or Kruskal Wallis test and categorical variables were analysed using chi-square test or Fisher’s exact test when appropriate

* p-value <0.05

**Table 2c. Healthcare Utilization of patients in 2013 and 3-year mortality data (k=4)**

| **Healthcare utilization / mortality by year^#^** | **Class 1**  Low comorbidity  **(n=64,091)** | **Class 2**  Moderate metabolic disease    **(n=32,389)** | **Class 3**  High metabolic and vascular disease  **(n=3,013)** | **Class 4**  High respiratory disease  **(n=1,254)** | **Overall**  **(n=100,747)** | **p-value** |
| --- | --- | --- | --- | --- | --- | --- |
| Number of primary care outpatient clinic visits, (SD) | 2.2 (3.9) | 5.8 (4.0) | 5.7 (6.5) | 5.0 (6.1) | 3.5 (4.4) | <0.001 |
| Number of outpatient specialist clinic visit, (SD) | 1.7 (4.7) | 2.7 (5.9) | 6.4 (9.8) | 4.0 (7.4) | 2.2 (5.5) | <0.001 |
| Number of hospital admission, (SD) | 0.1 (0.4) | 0.1 (0.5) | 0.7 (1.4) | 0.4 (1.0) | 0.1 (0.5) | <0.001 |
| Number of emergency department visits, (SD) | 0.1 (0.5) | 0.2 (0.6) | 0.7 (1.7) | 0.7 (2.6) | 0.1 (0.7) | <0.001 |
| 3 year all-cause mortality, (%) | 809 (1.3) | 1480 (4.6) | 713 (23.7) | 122 (9.7) | 3124 (3.1) | <0.001 |

**Abbreviations**: SB – standard deviation

^#^ Continuous variables were analyzed using Anova test or Kruskal Wallis test and categorical variables were analysed using chi-square test or Fisher’s exact test when appropriate

* p-value <0.05

**Table 2d. Healthcare Utilization of patients in 2013 and 3-year mortality data (k=5)**

| **Healthcare utilization / mortality by year^#^** | **Class 1**  Low comorbidity  **(n=61,792)** | **Class 2**  Moderate metabolic disease    **(n=24,225)** | **Class 3**  High metabolic and vascular disease  **(n=2,510)** | **Class 4**  High respiratory disease  **(n=1,209)** | **Class 5**  High metabolic disease without complication  **(n=11,011)** | **Overall**  **(n=100,747)** | **p-value** |
| --- | --- | --- | --- | --- | --- | --- | --- |
| Number of primary care outpatient clinic visits, (SD) | 2.1 (3.9) | 5.6 (4.0) | 5.4 (6.5) | 5.0 (6.2) | 6.2 (4.1) | 3.5 (4.4) | <0.001 |
| Number of outpatient specialist clinic visit, (SD) | 1.7 (4.6) | 3.0 (6.3) | 6.9 (10.2) | 4.0 (7.5) | 2.5 (5.5) | 2.2 (5.5) | <0.001 |
| Number of hospital admission, (SD) | 0.1 (0.3) | 0.1 (0.6) | 0.7 (1.5) | 0.4 (1.0) | 0.1 (0.5) | 0.1 (0.5) | <0.001 |
| Number of emergency department visits, (SD) | 0.1 (0.5) | 0.2 (0.6) | 0.8 (1.7) | 0.7 (2.7) | 0.2 (0.6) | 0.1 (0.7) | <0.001 |
| 3 year all-cause mortality, (%) | 740 (1.2) | 1033 (4.3) | 648 (25.8) | 113 (9.4) | 590 (5.4) | 3124 (3.1) | <0.001 |

**Abbreviations**: SB – standard deviation

^#^ Continuous variables were analyzed using Anova test or Kruskal Wallis test and categorical variables were analysed using chi-square test or Fisher’s exact test when appropriate

* p-value <0.05

**Table 3a. Multivariate negative binomial regression on healthcare utilization in Year 2013 and cox proportional hazards regression on 3-year all-cause mortality (k=2)**

| **Healthcare utilization or mortality** | **IRR, unless otherwise specified** | **95% Confidence interval** | **p-value** |
| --- | --- | --- | --- |
| **Number of primary care outpatient clinic visits** |  |  |  |
| Class 1 | 1.00 | *Reference* |  |
| Class 2 | 2.65 | 2.62 – 2.69 | <0.001 |
| **Number of outpatient specialist clinic visit** |  |  |  |
| Class 1 | 1.00 | *Reference* |  |
| Class 2 | 1.91 | 1.85 – 1.97 | <0.001 |
| **Number of hospital admission** |  |  |  |
| Class 1 | 1.00 | *Reference* |  |
| Class 2 | 2.88 | 2.73 – 3.05 | <0.001 |
| **Number of emergency department visits** |  |  |  |
| Class 1 | 1.00 | *Reference* |  |
| Class 2 | 2.39 | 2.27 – 2.51 | <0.001 |
| **All-cause mortality^#^** |  |  |  |
| Class 1 | 1.00 | *Reference* |  |
| Class 2 | 4.96 | 4.65 – 5.30 | <0.001 |

Abbreviations: IRR - Incidence rate ratio

# - Hazard ratio was reported

**Table 3b. Multivariate negative binomial regression on healthcare utilization in Year 2013 and cox proportional hazards regression on 3-year all-cause mortality (k=3)**

| **Healthcare utilization or mortality** | **IRR, unless otherwise specified** | **95% Confidence interval** | **p-value** |
| --- | --- | --- | --- |
| **Number of primary care outpatient clinic visits** |  |  |  |
| Class 1 | 1.00 | *Reference* |  |
| Class 2 | 2.65 | 2.61 – 2.69 | <0.001 |
| Class 3 | 2.82 | 2.72 – 2.92 | <0.001 |
| **Number of outpatient specialist clinic visit** |  |  |  |
| Class 1 | 1.00 | *Reference* |  |
| Class 2 | 1.65 | 1.60 – 1.70 | <0.001 |
| Class 3 | 4.43 | 4.10 – 4.78 | <0.001 |
| **Number of hospital admission** |  |  |  |
| Class 1 | 1.00 | *Reference* |  |
| Class 2 | 1.98 | 1.87 – 2.10 | <0.001 |
| Class 3 | 11.53 | 10.34 – 12.87 | <0.001 |
| **Number of emergency department visits** |  |  |  |
| Class 1 | 1.00 | *Reference* |  |
| Class 2 | 1.72 | 1.64 – 1.81 | <0.001 |
| Class 3 | 8.84 | 8.00 – 9.76 | <0.001 |
| **All-cause mortality^#^** |  |  |  |
| Class 1 | 1.00 | *Reference* |  |
| Class 2 | 3.57 | 3.34 – 3.83 | <0.001 |
| Class 3 | 18.48 | 17.03 – 20.05 | <0.001 |

Abbreviations: IRR - Incidence rate ratio

# - Hazard ratio was reported

**Table 3c. Multivariate negative binomial regression on healthcare utilization in Year 2013 and cox proportional hazards regression on 3-year all-cause mortality (k=4)**

| **Healthcare utilization or mortality** | **IRR, unless otherwise specified** | **95% Confidence interval** | **p-value** |
| --- | --- | --- | --- |
| **Number of primary care outpatient clinic visits** |  |  |  |
| Class 1 | 1.00 | *Reference* |  |
| Class 2 | 2.67 | 2.63 – 2.71 | <0.001 |
| Class 3 | 2.84 | 2.74 – 2.95 | <0.001 |
| Class 4 | 2.36 | 2.23 – 2.50 | <0.001 |
| **Number of outpatient specialist clinic visit** |  |  |  |
| Class 1 | 1.00 | *Reference* |  |
| Class 2 | 1.67 | 1.61 – 1.72 | <0.001 |
| Class 3 | 4.52 | 4.17 – 4.91 | <0.001 |
| Class 4 | 2.64 | 2.33 – 3.00 | <0.001 |
| **Number of hospital admission** |  |  |  |
| Class 1 | 1.00 | *Reference* |  |
| Class 2 | 2.03 | 1.91 – 2.14 | <0.001 |
| Class 3 | 12.30 | 10.96 – 13.79 | <0.001 |
| Class 4 | 5.53 | 4.61 – 6.64 | <0.001 |
| **Number of emergency department visits** |  |  |  |
| Class 1 | 1.00 | *Reference* |  |
| Class 2 | 1.81 | 1.72 – 1.90 | <0.001 |
| Class 3 | 9.24 | 8.33 – 10.26 | <0.001 |
| Class 4 | 7.45 | 6.38 – 8.70 | <0.001 |
| **All-cause mortality^#^** |  |  |  |
| Class 1 | 1.00 | *Reference* |  |
| Class 2 | 3.72 | 3.47 – 3.99 | <0.001 |
| Class 3 | 19.77 | 18.17 – 21.52 | <0.001 |
| Class 4 | 7.63 | 6.49 – 8.98 | <0.001 |

Abbreviations: IRR - Incidence rate ratio

# - Hazard ratio was reported

**Table 3d. Multivariate negative binomial regression on healthcare utilization in Year 2013 and cox proportional hazards regression on 3-year all-cause mortality (k=5)**

| **Healthcare utilization or mortality** | **IRR, unless otherwise specified** | **95% Confidence interval** | **p-value** |
| --- | --- | --- | --- |
| **Number of primary care outpatient clinic visits** |  |  |  |
| Class 1 | 1.00 | *Reference* |  |
| Class 2 | 2.69 | 2.65 – 2.73 | <0.001 |
| Class 3 | 2.88 | 2.77 – 3.00 | <0.001 |
| Class 4 | 2.48 | 2.34 – 2.63 | <0.001 |
| Class 5 | 2.98 | 2.92 – 3.04 | <0.001 |
| **Number of outpatient specialist clinic visit** |  |  |  |
| Class 1 | 1.00 | *Reference* |  |
| Class 2 | 1.84 | 1.78 – 1.91 | <0.001 |
| Class 3 | 5.05 | 4.61 – 5.52 | <0.001 |
| Class 4 | 2.72 | 2.39 – 3.09 | <0.001 |
| Class 5 | 1.59 | 1.52 -1.67 | <0.001 |
| **Number of hospital admission** |  |  |  |
| Class 1 | 1.00 | *Reference* |  |
| Class 2 | 2.16 | 2.03 – 2.30 | <0.001 |
| Class 3 | 14.34 | 12.67 – 16.22 | <0.001 |
| Class 4 | 5.70 | 4.73 – 6.86 | <0.001 |
| Class 5 | 2.24 | 2.06 – 2.42 | <0.001 |
| **Number of emergency department visits** |  |  |  |
| Class 1 | 1.00 | *Reference* |  |
| Class 2 | 1.95 | 1.85 – 2.06 | <0.001 |
| Class 3 | 10.62 | 9.50 – 11.88 | <0.001 |
| Class 4 | 7.76 | 6.63 – 9.08 | <0.001 |
| Class 5 | 1.93 | 1.79 – 2.07 | <0.001 |
| **All-cause mortality^#^** |  |  |  |
| Class 1 | 1.00 | *Reference* |  |
| Class 2 | 3.64 | 3.37 – 3.93 | <0.001 |
| Class 3 | 23.05 | 21.10 – 25.19 | <0.001 |
| Class 4 | 7.71 | 6.52 – 9.13 | <0.001 |
| Class 5 | 4.58 | 4.20 – 5.00 | <0.001 |

Abbreviations: IRR - Incidence rate ratio

# - Hazard ratio was reported

**Figure 2a. Kaplan Meier survival estimate by patient class (k=2)**

| **Days** | **0** | **365** | **730** | **1095** |
| --- | --- | --- | --- | --- |
| Number at Risk |  |  |  |  |
| Class 1: Low comorbidity | 65,012 | 64,849 | 64,641 | 64,452 |
| Class 2: High comorbidity | 35,735 | 35,338 | 34,785 | 34,225 |

Log-Rank Test: p<0.001

**Figure 2b. Kaplan Meier survival estimate by patient class (k=3)**

| **Days** | **0** | **365** | **730** | **1095** |
| --- | --- | --- | --- | --- |
| Number at Risk |  |  |  |  |
| Class 1: Low comorbidity | 64,813 | 64648 | 64435 | 64246 |
| Class 2: Moderate metabolic disease | 32,519 | 32295 | 31959 | 31584 |
| Class 3: High metabolic and vascular disease | 3,415 | 3244 | 3032 | 2847 |

Log-Rank Test: p<0.001

**Figure 2c. Kaplan Meier survival estimate by patient class (k=4)**

| **Days** | **0** | **365** | **730** | **1095** |
| --- | --- | --- | --- | --- |
| Number at Risk |  |  |  |  |
| Class 1: Low comorbidity | 64,091 | 63938 | 63733 | 63554 |
| Class 2: Moderate metabolic disease | 32,389 | 32171 | 31830 | 31460 |
| Class 3: High metabolic and vascular disease | 3,013 | 2860 | 2668 | 2500 |
| Class 4: High respiratory disease | 1,254 | 1218 | 1195 | 1163 |

Log-Rank Test: p<0.001

**Figure 2d. Kaplan Meier survival estimate by patient class (k=5)**

| **Days** | **0** | **365** | **730** | **1095** |
| --- | --- | --- | --- | --- |
| Number at Risk |  |  |  |  |
| Class 1: Low comorbidity | 61,792 | 61645 | 61463 | 61298 |
| Class 2: Moderate metabolic disease | 24,225 | 24070 | 23817 | 23566 |
| Class 3: High metabolic and vascular disease | 2,510 | 2375 | 2195 | 2038 |
| Class 4: High respiratory disease | 1,209 | 1175 | 1154 | 1124 |
| Class 5: High metabolic disease without complication | 11,011 | 10922 | 10797 | 10651 |

Log-Rank Test: p<0.001
